# A mixed methods evaluation of the maternal-newborn dashboard in Ontario: dashboard attributes, contextual factors, and facilitators and barriers to use: a study protocol

**DOI:** 10.1186/s13012-016-0427-1

**Published:** 2016-05-04

**Authors:** Sandra Dunn, Ann E. Sprague, Jeremy M. Grimshaw, Ian D. Graham, Monica Taljaard, Deshayne Fell, Wendy E. Peterson, Elizabeth Darling, JoAnn Harrold, Graeme N. Smith, Jessica Reszel, Andrea Lanes, Carolyn Truskoski, Jodi Wilding, Deborah Weiss, Mark Walker

**Affiliations:** 1 Better Outcomes Registry & Network (BORN Ontario), 401 Smyth Road, Ottawa, ON K1H 8 L1 Canada; 2 Department of Medicine, Ottawa Hospital Research Institute (OHRI), Clinical Epidemiology Program, University of Ottawa, 501 Smyth Road, Ottawa, ON K1H 8 L6 Canada; 3 Ottawa Hospital Research Institute (OHRI), School of Epidemiology, Public Health and Preventive Medicine, University of Ottawa, 501 Smyth Road, Ottawa, ON K1H 8 L6 Canada; 4 Ottawa Hospital Research Institute (OHRI), School of Epidemiology, Public Health and Preventive Medicine, University of Ottawa, 1053 Carling Avenue, Ottawa, ON K1Y 4E9 Canada; 5 School of Nursing, University of Ottawa, 451 Smyth Road, Ottawa, ON K1H 8 M5 Canada; 6 Laurentian University, 935 Ramsey Lake Road, Sudbury, ON P3E 2C6 Canada; 7 Children’s Hospital of Eastern Ontario (CHEO), 401 Smyth Road, Ottawa, ON K1H 8 L1 Canada; 8 Kingston General Hospital, 76 Stuart Street, Kingston, ON K7L 2 V7 Canada; 9 Better Outcomes Registry & Network (BORN Ontario), Children’s Hospital of Eastern Ontario (CHEO) Research Institute, 401 Smyth Road, Ottawa, ON K1H 8 L1 Canada; 10 Better Outcomes Registry & Network (BORN Ontario), School of Nursing, University of Ottawa, 401 Smyth Road, Ottawa, ON K1H 8 L1 Canada; 11 Ottawa Hospital Research Institute (OHRI), University of Ottawa, Better Outcomes Registry & Network (BORN Ontario), 501 Smyth Road, Ottawa, ON K1H 8 L6 Canada

**Keywords:** Audit and feedback, Dashboard, Maternal-newborn care, Knowledge translation, Maternal health services, Health-care quality assurance, Benchmarking, Mixed methods

## Abstract

**Background:**

There are wide variations in maternal-newborn care practices and outcomes across Ontario. To help institutions and care providers learn about their own performance, the Better Outcomes Registry & Network (BORN) Ontario has implemented an audit and feedback system, the Maternal-Newborn Dashboard (MND), for all hospitals providing maternal-newborn care. The dashboard provides (1) near real-time feedback, with site-specific and peer comparison data about six key performance indicators; (2) a visual display of evidence-practice gaps related to the indicators; and (3) benchmarks to provide direction for practice change. This study aims to evaluate the effects of the dashboard, dashboard attributes, contextual factors, and facilitation/support needs that influence the use of this audit and feedback system to improve performance. The objectives of this study are to (1) evaluate the effect of implementing the dashboard across Ontario; (2) explore factors that potentially explain differences in the use of the MND among hospitals; (3) measure factors potentially associated with differential effectiveness of the MND; and (4) identify factors that predict differences in hospital performance.

**Methods/design:**

A mixed methods design includes (1) an interrupted time series analysis to evaluate the effect of the intervention on six indicators, (2) key informant interviews with a purposeful sample of directors/managers from up to 20 maternal-newborn care hospitals to explore factors that influence the use of the dashboard, (3) a provincial survey of obstetrical directors/managers from all maternal-newborn hospitals in the province to measure factors that influence the use of the dashboard, and (4) a multivariable generalized linear mixed effects regression analysis of the indicators at each hospital to quantitatively evaluate the change in practice following implementation of the dashboard and to identify factors most predictive of use.

**Discussion:**

Study results will provide essential data to develop knowledge translation strategies for facilitating practice change, which can be further evaluated through a future cluster randomized trial.

## Background

**Fig. 1:**
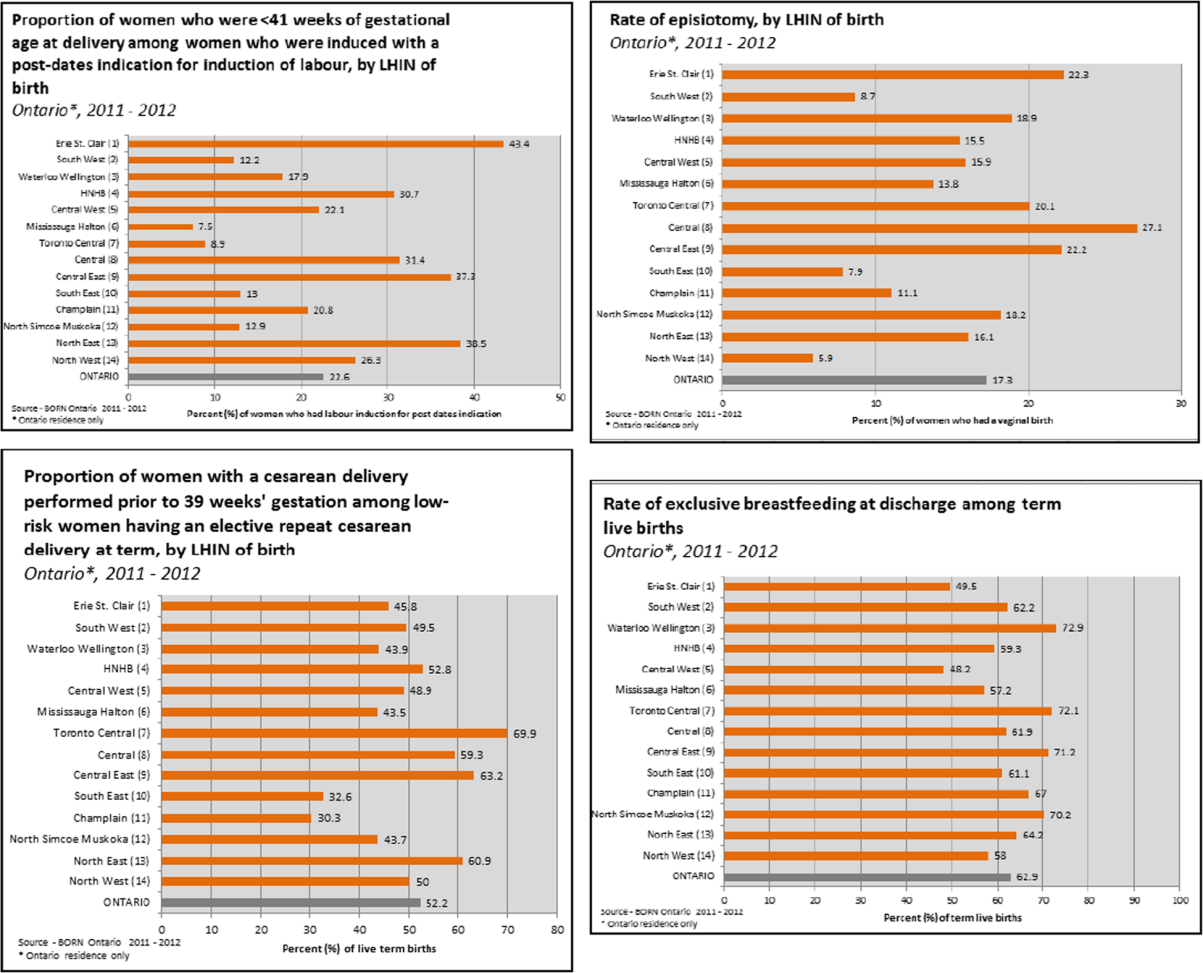
Examples of practice variation across Ontario for selected indicators

Pregnancy, labor, birth, and the early postpartum period are characterized by high utilization of health-care services, and outcomes from this period have important implications for current and future population health. There are approximately 140,000 babies born in Ontario hospitals each year [1]. Wide variation in maternal-newborn care practices and outcomes exists across Ontario indicating that optimal care is not always delivered and there are opportunities to address quality of care (see Fig. 1). To help institutions and care providers learn more about their own performance, the Better Outcomes Registry & Network (BORN) Ontario implemented the Maternal-Newborn Dashboard (MND) [2], an audit and feedback (A&F) system for all maternal-newborn hospitals in Ontario.

**Fig. 2:**
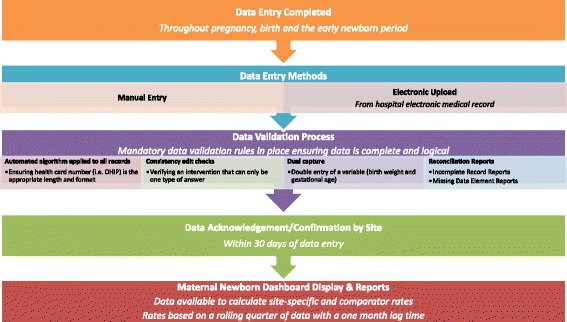
BORN data entry and validation process flow chart for hospital birth data

BORN Ontario is a prescribed registry under Ontario Personal Health Information Protection Act (PHIPA) privacy legislation and is able to collect, disclose, and use personal health information for the purpose of improving care and patient outcomes. The BORN Information System (BIS), an Internet-based data collection system, is operational in all hospitals providing maternal-newborn care and has data for all hospital births. Maternal demographics and health behaviors, pre-existing maternal health problems, obstetric complications, intrapartum interventions, and birth and newborn outcomes are captured at the time of birth from medical records, clinical forms, and patient interviews. These data are either entered into the BIS by hospital staff or uploaded directly from hospitals’ electronic health records. Each site has access to their own data, and BORN Ontario reports on outcomes aggregated at the provincial level at regular intervals [1]. An ongoing data validation process [3], quality checks, and formal training sessions for individuals entering data assure a high level of data quality (see Fig. 2). A number of papers have been published using these data [1, 4–6].

The MND is an A&F reporting tool within the BIS and was launched in November 2012. The MND targets six key performance indicators (KPIs), which were identified during the rigorous development process [2] (see Table 1).

**Table 1:** BORN maternal-newborn dashboard key performance indicators, definitions, and benchmarks

Key performance indicators	Target (green)	Warning (yellow)	Alert (red)	Definitions
	%	%	%	
1. Proportion of newborn screening samples that are unsatisfactory for testing	<2.0	2.0–3.0	>3.0	Number of newborn screening samples with unsatisfactory reason(s), expressed as a percentage of the total number of newborn screening samples submitted from a given hospital/midwifery practice. NOTE: Samples coded as unsatisfactory only due to collection at less than 24 h of age (i.e., there are no other reasons the sample has been deemed unsatisfactory) will not be considered as unsatisfactory for this analysis, since sample collection at less than 24 h of age is recommended in cases of early discharge, transfer, or transfusion.
2. Proportion of episiotomy in spontaneous vaginal births	<13.0	13.0–17.0	>17.0	Number of women having a spontaneous vaginal birth who have an episiotomy expressed as a percentage of the total number of women having a spontaneous vaginal birth (in a given place and time).
3. Proportion of formula supplementation in term infants whose mothers intended to breastfeed	<20.0	20.0–25.0	>25.0	Number of term live babies receiving formula supplementation expressed as a percentage of the total number of term babies whose mothers intended to breastfeed (in a given place and time).
4. Proportion of repeat cesarean section in low-risk women not in labor at term with no medical or obstetrical complications done prior to 39-week gestation	<11.0	11.0–15.0	>15.0	Number of women with a cesarean section performed from >37 to <39-week gestation, expressed as a percentage of the total number of low-risk women having a repeat cesarean section at term (in a given place and time). NOTE: Repeat cesarean delivery in low-risk women is defined as a cesarean section performed prior to the onset of labor, among women with a singleton live birth, with a history of one or more previous cesarean sections and with no fetal or maternal health conditions or obstetrical complications. Women with indication for cesarean section are excluded, other than the following indications: fetal malposition/malpresentation, previous cesarean section, accommodates care provider/organization, maternal request.
5. Proportion of women delivering at term who had GBS screening at 35–37-week gestation	>94.0	90.0–94.0	<90.0	Number of women having an unplanned cesarean section who deliver at term and have GBS screening at 35–37-week gestation expressed as a percentage of the total number of laboring women delivering at term (given place and time).
6. Proportion of women induced with an indication of post-dates who are less than 41-week gestation at delivery	<5.0	5.0–10.0	>10.0	Number of women who were less than 41 weeks of gestation at delivery, expressed as a percentage of the total number of women who had labor induction and an indication for induction of “post‐dates pregnancy” (given place and time).

For A&F to be effective, it must be timely, individualized, non-punitive, customizable, and perceived as relevant and credible [7–9]. A rigorous development process was undertaken to ensure credibility of the BORN Ontario MND [2]. First, the framework for selecting core quality measures (clinically meaningful, feasible to monitor, and actionable/amenable to change) was used to guide selection of the KPIs for the MND and allowed us to identify a manageable number of indicators [2]. We then validated the six potential indicators as appropriate for use across the province by assessing current performance of these indicators across the 14 health regions in Ontario. Simultaneously, evidence summaries for each of the potential indicators were developed in collaboration with the Knowledge to Action Research Centre at the Ottawa Hospital Research Institute [10–14] (http://bornontario.ca/en/born-information-system/report-training/). To establish benchmarks for the KPIs, we used a combination of peer-reviewed literature, current clinical practice within Ontario, and recommendations from clinical experts. Glantz’s recommendation to accept lower intervention rates if higher rates do not improve outcomes was a guiding principle [15], as well as other risk assessment techniques such as restricting patient populations to more homogeneous subgroups.

**Fig. 3:**
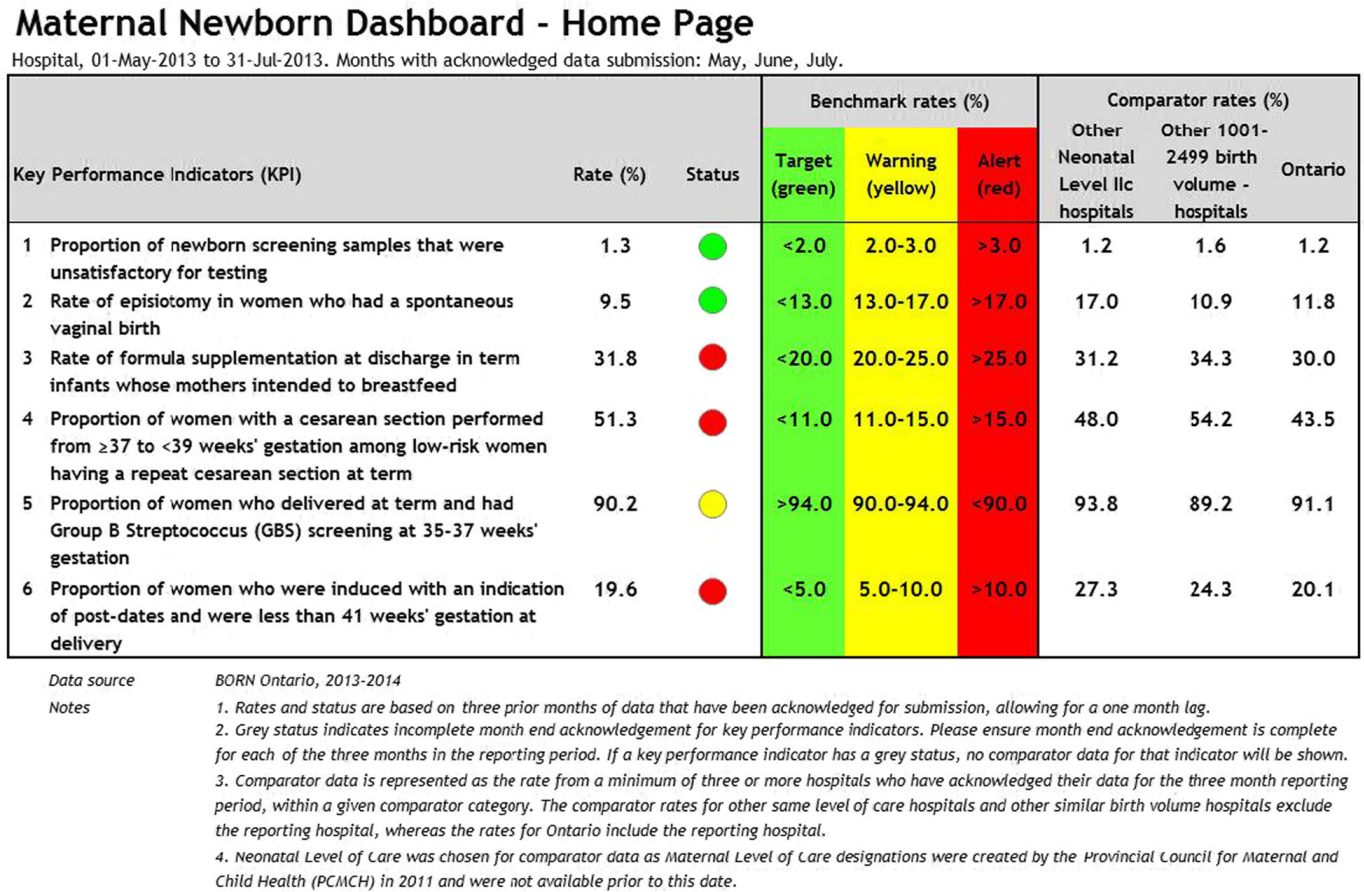
BORN maternal-newborn dashboard sample home page

The BORN MND report provides users with (1) near real-time feedback, site-specific, and peer comparison data for the aforementioned six KPIs; (2) a signal or visual display of evidence-practice gaps related to the KPIs; and (3) benchmarks to provide direction for practice change (see Fig. 3). The ultimate goal is to promote evidence-informed practice, decrease variability in care processes related to the selected KPIs, and improve maternal-newborn outcomes.

Awareness of practice issues and agreement about the evidence and need for change are essential first steps for adoption of new care practices [16]. This suggests that increasing practitioner awareness about a problem (e.g., by providing feedback about KPI rates) will facilitate engagement in practice change, reduce practice variation, and lead to improved patient care. In 2010, we conducted a pilot project in one health region in Ontario to evaluate the effectiveness of the A&F process using an early version of the MND [4]. This feasibility study proved successful in reducing the rate of elective repeat Caesarian section < 39 weeks' gestation in the region and suggested that the MND may be a useful tool to facilitate practice change across the province [4].

With the implementation of the new MND system, BORN Ontario is in a unique position to evaluate this A&F tool. Dashboards have been used for a variety of purposes within health care but are primarily implemented to drive quality improvement. The use of a dashboard may improve quality of care and patient outcomes [17–20]. A Cochrane review of 140 randomized controlled trials demonstrated small but important changes in practice [9], and the universal use of a maternity dashboard has been strongly recommended in the UK to improve clinical practice [21]. However, it is unclear why performance improves in some settings and not in others, what resources are needed to increase the effectiveness of a system like the MND, and which attributes of electronic A&F have the greatest effect. We plan to address this issue by evaluating the effect of the MND among hospitals in Ontario to facilitate practice change for selected KPIs for maternal-newborn care and explore factors predicting variation in response.

### Goal and objectives

The overall goals of this mixed methods study are to increase our knowledge of how A&F works in hospital settings and to support the design of cluster randomized trials according to the Medical Research Council-recommended framework for evaluation of complex interventions [22].

More specifically, the study aims to:

1. Conduct an interrupted time series (ITS) analysis to evaluate the population effect of implementing the MND across Ontario by comparing hospital performance on six selected KPIs
2. Qualitatively explore factors that potentially explain differences in the use of the MND among hospitals by conducting key informant interviews with directors/managers from a diverse group of maternal-newborn hospitals in Ontario
3. Measure factors potentially associated with differential effectiveness of the MND by developing and implementing a survey of all maternal-newborn hospitals in Ontario
4. Identify factors significantly associated with differences in hospital performance before and after implementation of the MND by conducting a longitudinal multivariable regression analysis of six KPIs

## Methods/design

### Design

This mixed methods study will use quantitative (objectives 1, 3, and 4) and qualitative (objective 2) methodologies to address the research objectives. We will implement a sequential exploratory design by using the qualitative findings (from interviews) to inform the quantitative components of the study (survey and regression analysis) [23].

### Theoretical framework

The Promoting Action on Research Implementation in Health Services (PARiHS) [24] framework will guide this study. The PARiHS framework contends that *evidence*, *context*, and *facilitation* are the key influencing factors for successful implementation of a new innovation for practice change. The most successful implementation occurs when evidence is scientifically robust and matches professional consensus and patient preferences (high evidence), when the context is receptive for change with sympathetic cultures, strong leadership, and appropriate monitoring and feedback systems (high context), and when there is appropriate facilitation of change, with input from skilled external and internal facilitators (high facilitation) [24]. Based on this framework, we hypothesize that successful adoption of the MND (a new innovation) to facilitate practice change will be dependent upon the nature and the clarity of the evidence on which it is based, the quality of the context (environment) in which it is being implemented, and the type of facilitation needed to ensure successful adoption. Concepts from the PARiHS framework will inform development of our semi-structured interview guide and the provincial survey for this study.

A description of the methods for each of the objectives is presented below.

### Objective 1—to evaluate the population effect of implementing the MND across Ontario

#### Methods

An ITS analysis of six KPIs (Table 1) will be used. This quasi-experimental design can be used to determine the effect of a complex intervention introduced at a specific point in time [25]. It is superior to many other quasi-experimental and observational designs, such as before and after designs, in that it avoids threats to internal validity such as history and maturation [26, 27]. By using outcomes assessed at multiple time points, it allows the estimation of an underlying secular trend prior to the intervention. The estimated intercept and trend before and after the intervention can then be compared to determine both the immediate and long-term effects of the intervention. While ITS designs are useful for determining whether an intervention has had an effect on the outcome while accounting for any underlying secular trend, the possibility of confounding by temporally concurrent interventions still poses an important threat to the internal validity of this design [28]. We will use two approaches to assess “possible rival explanations” for any apparent effect of the MND [29]. First, we will assess two non-equivalent outcomes measured in the same population over the same period of time: CS rates in nulliparous women with induced labor and the proportion of low-risk women at term gestation having electronic fetal monitoring in labor. Since these outcomes should not be affected by the MND, a finding of no change in these variables will strengthen our ability to attribute any change in the KPIs to the MND. Second, we will use data from two Canadian provinces (Nova Scotia and British Columbia) as a non-equivalent control group. A suitable control group must have similar baseline characteristics and pre-implementation temporal trends, except for the MND intervention [30].

#### Analysis

The data to support the primary ITS analysis will come from the BIS. The MND implementation occurred from November 2012 to March 2013, allowing time for all hospitals to gain experience with data entry and use of the dashboard. MND data for the 3-year pre-implementation period (November 2009–October 2012) and the 2-year post-implementation period (April 2013–March 2015) will be available after April 2016. Thus, each site will contribute 3 years of data to the pre-implementation phase and 2 years after implementation. We will exclude data from the 5–6 months between the pre- and post-implementation phases from the analysis. For our primary ITS analyses, the six KPIs will be expressed as monthly proportions after pooling across all hospitals. Pooling is necessary to allow inclusion of low birth volume hospitals and to accommodate all six KPIs, including two that assess performance of uncommon outcomes. Time series plots will be used to visually inspect the immediate and long-term effect of the intervention and the presence of trends, cyclical patterns, and outliers. A segmented regression analysis will be completed using separate intercepts and slopes for the time periods before and after implementation of the MND [26, 31]. The presence of autocorrelation will be assessed using Durbin-Watson tests, as well as visual inspection of residual plots. If autocorrelation is present, an autocorrelation parameter will be included in the model. The results of the segmented regression analysis of each KPI will be reported as level and trend changes after the intervention, with 95 % confidence intervals (CI). All analyses will be conducted using SAS v. 9.4.

#### Sample size considerations

The ITS analysis will include monthly data aggregated from 94 hospitals over 5 years (60 time points total). Recommendations are to include between 40 and 50 observations for robust statistical analysis of ITS designs [32]; moreover, to avoid over-fitting of segmented regression models, at least 10 observations for each regression coefficient is required [33]. With 4 regression coefficients in our planned analyses, 60 time points is adequate. Furthermore, to help ensure stability of the monthly proportions, it is recommended that measurements be based on at least 100 observations at each time point. After pooling across all hospitals (approximately 12,000 births per month), the minimum monthly denominator will range from approximately 200 (repeat Caesarian section) to 7800 (group B Streptococcus screening). Thus, we expect to have sufficient sample sizes to ensure stable estimates.

### Objective 2—to explore factors that potentially explain differences among hospitals’ use of the MND

#### Methods

##### Hospital selection

A criterion-based approach [34] will be used to identify a purposeful sample of up to 20 hospitals reflecting different levels of care (levels 1, 2, and 3) [35], annual birth volumes (<1000, 1001–2500, >2500), geographic locations, and degree of engagement with the MND (i.e., none, partial, or full). This approach to hospital selection will provide a diverse sample of hospitals from which to recruit participants for the interviews and ensure a rich source of data to inform our understanding of factors potentially associated with differential effectiveness of the MND.

##### Sampling procedures

In qualitative research, there are no standardized rules for sample sizes: while 6–8 participants often suffice for a homogeneous sample, 20–30 may be needed when looking for disconfirming evidence or trying to achieve maximum variation [36, 37]. We will use the concept of data saturation to determine when no additional interviews are required (i.e., no new information is emerging) [38, 39]. Directors or managers of the maternal-newborn units from up to 20 of the purposefully selected hospitals (key informants) will be invited to participate in an interview. Recruitment may be augmented through snowball sampling. We do not anticipate problems recruiting participants because of our extensive connections with these centers. Following ethical approval, we will identify individuals within each organization willing to participate, who can provide information regarding use of the MND with regard to its utility as an A&F tool for practice change. Participants will be recruited based on their familiarity with the BIS, their ability to describe practice from the perspective of the organization, their knowledge of the KPIs in the MND, and quality improvement within their organization. Participant consent will be obtained prior to scheduling the interviews.

##### Semi-structured interview guide

Key informant interviews will be completed using a semi-structured interview guide. Interview questions will be based on the concepts in the PARiHS framework and the Organizational Readiness for Knowledge Translation (OR4KT) Tool [40, 41], a comprehensive evidence-based instrument that was developed based on a systematic review of conceptual models/frameworks of organizational readiness for change in health care. The OR4KT has been validated in primary care settings and contains questions covering six dimensions of organizational readiness (organizational climate for change, organizational contextual factors, change content, leadership, organizational support, and motivation). The interview questions will be designed to probe participants’ perspectives about the attributes of the MND, hospital contextual factors, and facilitation/support issues that have influenced their hospital’s use of the MND. Interviews, which may last up to 1 h, will be conducted in person or by telephone and will be audiotaped, with consent. The interview guide will be pilot tested and questions revised if necessary.

##### Data entry and processing

Interviewing, transcription, and analysis will proceed concurrently to monitor the progress of the interviews, permit follow-up of issues that may emerge from the data, and allow probing of emerging themes in subsequent interviews [42, 43]. Digital recordings will be transcribed verbatim and verified by the interviewer prior to analysis. Data will be imported into NVivo 11™ (qualitative data management software) to facilitate management of data analysis [44].

#### Analysis

##### Within-case analysis

Initially, data from each of the cases (hospitals) will be analyzed independently. Analysis will begin with repeated reading of transcripts and field notes, summarizing key information by writing a description of each transcript [34, 45], followed by qualitative content analysis using coding, categorizing, and thematic description [43, 46, 47]. Codes will be sorted into (1) a priori categories based on PARiHS concepts (MND attributes, OR4KT dimensions [40, 41], and facilitation factors) and (2) categories that emerge during the analysis [34]. The final step in this within-case analysis will be the development of narrative descriptions of themes derived from each case. Member checking will be undertaken involving a small subgroup of participants to ensure that the themes identified through the coding process resonate with the participants’ experiences and to identify any gaps in the analysis or issues requiring further consideration [34, 48]*.*

##### Cross-case analysis

Subsequently, thematic similarities and differences between cases (based on hospital selection criteria) will provide understanding of the key factors influencing the use of the MND in different practice settings. Investigators will regularly discuss the coding template, categories, and emerging themes to build consensus regarding study findings. The findings will be used to generate hypotheses about factors that explain variability in performance after implementation of the MND and to inform development of a survey to measure these factors in all maternal-newborn hospitals in Ontario.

### Objective 3—to measure factors hypothesized to be associated with differential effectiveness of the MND

#### Methods

##### Sampling procedures

For each maternal-newborn hospital in the province, an individual knowledgeable about organizational structure, quality improvement, and clinical practice, such as the obstetrical director, will be invited to complete the survey. Following ethical approval, we will initiate contact with these potential respondents by email and provide information about the purpose of the study, how the survey results might be used, the confidentiality of the data, and an invitation to participate.

##### Questionnaire development

Information obtained from the key informant interviews (objective 2), concepts contained in the PARiHS Framework, and the OR4KT Tool will inform development of the provincial survey. The survey will be developed using REDCap (Research Electronic Data Capture), a secure, web-based application designed to support data capture for research studies, hosted at the Children’s Hospital of Eastern Ontario Research Institute (CHEO RI) [49]. The survey will have four components (demographic information, questions about the attributes of the MND, clinical behaviors related to the KPIs, and facilitation/user support needs, and the OR4KT questions). The OR4KT questions will be used to explore contextual factors potentially influencing effective use of the MND. New questions will be developed to probe participants’ perspectives about the attributes of the MND and clinical behaviors related to the KPIs (e.g., content and clarity of the information displayed in the MND, evidence supporting each KPI and benchmark, audit features and functionality, and user access). In addition, questions probing the concept of facilitation will be developed focusing on the intensity of the facilitation activities undertaken, satisfaction, and internal and external supports. We will pilot test the survey with clinicians and administrators for clarity, length, and flow of questions, and the questionnaire will be revised if necessary. The OR4KT Tool will be used in its entirety as designed.

##### Survey administration

To promote a high response rate, the survey will be designed and administered using Dillman’s Tailored Design Method [50] for electronic mail surveys. If the response rate has not reached 80 % by weeks 10–12 after reminders, a follow-up phone call will occur.

#### Analysis

Descriptive statistics will be used to summarize characteristics and factors measured in the survey. A list of 18–20 factors will be identified for further statistical analysis (objective 4). To assess the representativeness of the survey, differences in the characteristics of hospitals with and without responses will be investigated using chi-squared and two-sample *t* tests (or non-parametric tests where required).

### Objective 4—to identify factors significantly associated with differences in hospital performance before and after implementation of the MND

#### Analysis

We will conduct a multivariable generalized linear mixed-effects regression analysis of the repeated indicators at each hospital to identify those factors that are most predictive of between-hospital differences in effectiveness of the MND. Unlike the pooled analysis for objective 1, this analysis will use individual hospital-level data. The analysis will be limited to hospitals with annual birth volumes >100 to avoid numerical instability due to small denominators. With an anticipated 60 % response rate for the survey, approximately 50 hospitals will be included in the analysis. Appropriate time intervals for these longitudinal analyses will be chosen to avoid instability due to low denominators: we anticipate that 4 KPIs will be analyzed using quarterly intervals (12 pre-implementation and 8 post-implementation time points) and 2 KPIs will be analyzed using annual intervals (3 pre-implementation and 2 post-implementation time points). The generalized linear mixed-effects regression analysis of the repeated proportions at each hospital will use either a log-link function with the denominator specified as an offset term or a logit link function with the outcome specified in binomial form. For quarterly measurements, time will be modeled using a semi-parametric spline function with knots separating the pre- and post-implementation phases. Random intercepts and slopes will be specified for each hospital, and hospital-specific trends will be estimated using empirical Bayes’ best linear unbiased predictors. An advantage of this model is that estimates can be obtained even for smaller hospitals, as these estimates “borrow information” from the rest of the data, resulting in individual means that are shrunken towards the population mean. For annual measurements, time will be analyzed as a categorical variable and random intercepts will be specified for each hospital. To identify factors associated with differences among hospital trends over time, the candidate predictor variables identified in the survey related to the attributes of the MND (e.g., clarity of the KPI definitions, evidence summaries, visual displays, and audit features), contextual factors (e.g., leadership, culture, formal and informal interactions, and resources), and facilitation factors (e.g., training, resources, internal and external supports) will be entered into the model, together with their product interaction terms with time. To reduce the potential number of coefficients (2 intercepts and 2 slopes, 18–20 candidate predictors, 54–60 interaction terms), we will enter each candidate predictor variable plus its interactions with time separately into the model. Only those factors that have significant main or interaction effects will be considered for the full multivariable model. To arrive at a more parsimonious final model, we will use stepwise backward elimination, first removing interaction terms as necessary and then main effects.

### Study status

At the time of preparing this manuscript, we have hired research staff, obtained ethical approval, developed tools to classify hospitals, developed and piloted data collection tools, and begun the process of data collection for the ITS and provincial survey.

## Discussion

### Strengths and limitations

A major strength of our study is the use of a mixed methods approach to obtain more in-depth understanding of factors that influence use of an A&F system to improve performance. Additional strengths include the use of a theoretical framework (PARiHS) and a validated instrument (OR4KT) to guide data collection and analysis for objectives 2 (interviews) and 3 (provincial survey). We will have a large and inclusive sample size—all 94 maternal-newborn hospitals in Ontario for objectives 1 (ITS) and 3 (provincial survey), and therefore, we have the unique opportunity to see a wide variety of practice patterns and to examine use of the MND at a health system level, across diverse organizations and levels of care. We anticipate our project will provide important information to health-care funders and legislators, hospital administrators, and health-care providers. We will also use the findings from this collection of studies to support the development of a series of cluster randomized trials to test how to best to implement A&F in the future.

Study limitations include (1) exclusion of home births as the MND was customized for hospital birth performance issues; however, this group accounts for less than 2 % of all births in the province. Hospital births by midwives will be included; (2) any observed performance change could be due to factors other than the MND implementation; however, we will implement several strategies to allow us to quantitatively assess whether this may be the case; (3) a small number of hospitals will be excluded from the multivariable regression analysis due to low birth volumes; however, all remaining hospitals will be included in multivariable regression analyses to identify predictor variables; and (4) there is a risk of non-response bias in the provincial survey; however, BORN Ontario works within a network of all maternal-newborn care organizations in the province and has a well-established communication network established with health-care providers working within these settings, which will facilitate recruitment and data collection.

### Relevance, impact, and urgency of this study

Wide variation in maternal-newborn care practices and outcomes across Ontario indicates that optimal care is not always delivered [1]. BORN Ontario has developed a unique online A&F system in Canada to facilitate uptake of evidence to practice on a large scale. Although a number of characteristics of organizational readiness for change have been reported [51, 52] (e.g., leadership, resources, situational factors, culture and values, policies and procedures), we have the opportunity to augment this knowledge base and determine to what extent organizations with an effective data capture system vary in their readiness and capability to undertake change for enhancing quality care. This study will explore factors that influence effective use of an electronic A&F system and how organizations respond when warning signals occur. Since the protocol was developed, additional evidence [8] has been published emphasizing the need for further research on A&F. The MND provides us with a unique environment for knowledge translation science and the opportunity to study A&F within the context of a large population of maternal-newborn care providers, births, and hospitals with different levels of care and birth volumes. This will increase our understanding about what makes A&F more or less effective and what contextual and facilitation factors are most important for successful implementation of the MND to improve practice.

If this study demonstrates increased use and effectiveness of the MND to improve practice related to some or all of the KPIs, we anticipate several opportunities to improve on its design as well as to identify key organizational and facilitation factors predictive of increased use of an A&F system. This information will help to increase understanding about when, for whom, in what way and why A&F works in some settings and not others. First, it could provide information to improve the design features for A&F systems targeting organization level indicators (e.g., different data tables, graphs to display comparator data or trends over time, custom query features to allow sites to tailor displays to suit their organizational needs). Second, it could help to identify the most important factors that influence organizations’ readiness for change and resources that could be developed to support change related to various KPIs. Third, it could increase awareness about key issues to consider when selecting indicators to target provincial level quality improvement initiatives. Providing local experts with the tools to identify evidence-practice gaps, initiate practice change, and tailor strategies to effectively counter the barriers identified may improve the uptake of best evidence and the sustainability of practice change. This study addresses some of the evidence gaps found in the literature about A&F and as a naturalistic study embedded within a provincial context of maternal-newborn care, we have the potential to improve knowledge about the effectiveness of A&F to improve care. We have a wealth of data available in the BIS and a mandate to use that data to facilitate care where possible. The use of A&F to increase health-care provider awareness about evidence-practice gaps and to trigger quality improvement initiatives is the first step to improve care and ultimately patient outcomes.

## Ethics approval and consent to participate

The Children’s Hospital of Eastern Ontario Research Ethics Board (REB) has reviewed and approved this study (protocol #13/218X). In addition, REB approval has been received from the University of Ottawa (A01-14-03). Informed consent will be obtained from all participants. All data collection and reporting will be compliant with the Canadian privacy laws and Ontario PHIPA legislation regulating the use of BORN Ontario Registry data. Data will be securely stored at the BORN Ontario offices, Centre for Practice Changing Research Building, 401 Smyth Road, Children’s Hospital of Eastern Ontario, Ottawa, Ontario. All study records and documents will be stored for 7 years after the study has ended.

### Consent for publication

Not applicable.

### Availability of data and material

The research described here is being conducted at Better Outcomes Registry & Network (BORN) Ontario, a provincial maternal-child registry. BORN Ontario holds copies of province-wide databases that contain personal health information, and the record-level data cannot be publicly shared, according to the privacy laws in Ontario. Access to aggregated de-identified data on which the conclusions rely will be available, on request, from BORN Ontario. For future publications reporting results, aggregated data tables will be provided as supplementary files where possible.

## Abbreviations

A&F: audit and feedback
BIS: BORN Information System
BORN: Better Outcomes Registry & Network Ontario
CIHR: Canadian Institutes of Health Research
ITS: interrupted time series
KPI: key performance indicator
MND: maternal-newborn dashboard
OR4KT: Organization Readiness for Knowledge Translation
PARiHS: Promoting Action on Research Implementation in Health Services
PHIPA: Personal Health Information Protection Act
